# *TP53* mutations are associated with primary endocrine resistance in luminal early breast cancer

**DOI:** 10.1002/cam4.4376

**Published:** 2021-11-14

**Authors:** Isabel Grote, Stephan Bartels, Leonie Kandt, Laura Bollmann, Henriette Christgen, Malte Gronewold, Mieke Raap, Ulrich Lehmann, Oleg Gluz, Ulrike Nitz, Sherko Kuemmel, Christine zu Eulenburg, Michael Braun, Bahriye Aktas, Eva‐Maria Grischke, Claudia Schumacher, Kerstin Luedtke‐Heckenkamp, Ronald Kates, Rachel Wuerstlein, Monika Graeser, Nadia Harbeck, Matthias Christgen, Hans Kreipe

**Affiliations:** ^1^ Hannover Medical School Institute of Pathology Hannover Germany; ^2^ West German Study Group Moenchengladbach Germany; ^3^ Ev. Bethesda Hospital Moenchengladbach Germany; ^4^ University Clinics Cologne Women’s Clinic and Breast Center Cologne Germany; ^5^ Clinics Essen‐Mitte Breast Unit Essen Germany; ^6^ Charité Women’s Clinic Berlin Germany; ^7^ Clinics Rotkreuz, Breast Center Munich Germany; ^8^ University Clinics Essen Women’s Clinic Essen Germany; ^9^ University Clinics Leipzig Women’s Clinic Leipzig Germany; ^10^ University Clinics Tuebingen Women’s Clinic Tuebingen Germany; ^11^ St. Elisabeth Hospital Cologne Germany; ^12^ Niels Stensen Clinics Clinics for Oncology Osnabrueck Germany; ^13^ Department OB&GYN and CCC Munich LMU University Hospital Breast Center Munich Germany; ^14^ Department of Gynecology University Medical Center Hamburg Hamburg Germany

**Keywords:** breast cancer, endocrine proliferative response, Ki67, preoperative endocrine therapy, *TP53*

## Abstract

**Background:**

Whereas the genomic landscape of endocrine‐resistant breast cancer has been intensely characterized in previously treated cases with local or distant recurrence, comparably little is known about genomic alterations conveying primary non‐responsiveness to endocrine treatment in luminal early breast cancer.

**Methods:**

In this study, 622 estrogen receptor‐expressing breast cancer cases treated with short‐term preoperative endocrine therapy (pET) from the WSG‐ADAPT trial (NCT01779206) were analyzed for genetic alterations associated with impaired endocrine proliferative response (EPR) to 3‐week pET with tamoxifen or aromatase inhibitors. EPR was categorized as optimal (post‐pET Ki67 <10%) versus slightly, moderately, and severely impaired (post‐pET Ki67 10%–19%, 20%–34%, and ≥35%, respectively). Recently described gene mutations frequently found in previously treated advanced breast cancer were analyzed (*ARID1A*, *BRAF*, *ERBB2*, *ESR1*, *GATA3*, *HRAS*, *KRAS*, *NRAS*, *PIK3CA*, and *TP53*) by next‐generation sequencing. Amplifications of *CCND1*, *FGFR1*, *ERBB2*, and *PAK1* were determined by digital PCR or fluorescence in situ hybridization.

**Results:**

*ERBB2* amplification (*p* = 0.0015) and mutations of *TP53* (*p* < 0.0001) were significantly associated with impaired EPR. Impaired EPR in *TP53*‐mutated breast cancer cases was independent from the Oncotype DX Recurrence Score group and was seen both with tamoxifen‐ and aromatase inhibitor‐based pET (*p* = 0.0005 each).

**Conclusion:**

We conclude that impaired EPR to pET is suitable to identify cases with primary endocrine resistance in early luminal breast cancer and that *TP53*‐mutated luminal cancers might not be sufficiently treated by endocrine therapy alone.

## Graphical abstract

Early recognition of endocrine resistance could be enabled by impaired suppression of proliferation after short‐term preoperative endocrine therapy (pET). Impaired endocrine proliferative response (EPR, post‐pET Ki67 ≥10%) sorts out cancers carrying *TP53* mutations.

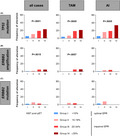

## 1 INTRODUCTION

About 75% of breast cancers (BCs) belong to the luminal type with estrogen‐depending tumor cell growth. Hormonal blockade with tamoxifen or aromatase inhibitor (AI) therefore provides an effective growth suppressive therapy for the majority of BCs. However, in a considerable proportion of luminal BC, that is, about 20%–30%, local or distant recurrence during or after endocrine treatment indicate resistance to endocrine therapy.^1^

Two general patterns of endocrine therapy resistance are recognized clinically: primary, intrinsic resistance, whereby estrogen receptor‐expressing (ER‐positive) cancers never adequately respond to endocrine treatment, and secondary, acquired resistance, which develops following an initial response.^2^ A major mechanism of acquired secondary resistance is provided by activating mutations in the ERα gene (*ESR1*).^3^ Over 30% of long‐term treated luminal BC display *ESR1* mutations. *ESR1* mutations are enriched in metastatic BC and with significant differences in metastatic site.^4^, ^5^ In bone metastasis, 14% of ER‐positive cases were found to be *ESR1*‐mutated.^6^ Mechanisms of intrinsic resistance may partly overlap but are currently far from being understood.

Efforts have been undertaken to identify the genetic characteristics of endocrine‐resistant BC.^1^ Razavi et al. analyzed 1501 luminal BC cases with resistance to endocrine therapy by next‐generation sequencing.^7^ The majority of cases in this study (87.5%) had been exposed to prior therapy in the adjuvant and/or metastatic setting.^7^ The number of treatment‐naïve cases was too small to allow a sufficiently powered analysis of primary genetic alterations. Genetic alterations occurring in more than 5% of metastases in this study included mutations of *ARID1A*, *ESR1*, *ERBB2*, and *TP53*.^7^

*TP53* gene mutation is frequent in the triple‐negative BC with up to 80% of cases showing this mutation.^7^, ^8^ In luminal BC, *TP53* mutation is encountered in about 12%–29% of cases.^8^ There appears to be an association with the luminal B phenotype.^9^, ^10^, ^11^ The majority of *TP53* mutations are somatic because selection for familial BC cases yielded lower proportions of *TP53*‐mutated cases indicating that germ‐line mutations (Li‐Fraumeni syndrome) are considerably rarer than somatic changes.^12^ Among different somatic mutations, *TP53* alterations were most frequent in metastatic luminal BC (29%).^13^ Whether *TP53* mutations evolve in the context of clonal evolution in recurring BC or are acquired during progression is not clear.^14^ In therapy‐naïve primary metastatic BC, *TP53* mutation was found in primary tumors as well as in metastatic deposits.^15^ Previous studies on the role of *TP53* in BC treatment response and survival have been summarized as contradictory and inconclusive.^16^, ^17^, ^18^

Neoadjuvant therapy of BC has generated new endpoints to evaluate therapy efficacy. In triple‐negative and HER2‐positive early BC, complete pathological remission after neoadjuvant therapy is commonly used as a surrogate marker for therapy responsiveness and favorable prognosis. In 70%–80% of BCs, this surrogate marker is not readily available because ER‐positive, luminal BCs will usually not regress completely when exposed even to long‐term preoperative endocrine therapy. Endocrine responsiveness, however, is indicated very precisely by a decrease in tumor cell proliferation, even after a short‐term exposure of only 2–3 weeks of endocrine therapy.^19^, ^20^, ^21^ The proliferation response in vivo can be assessed by the proliferation marker Ki67 and has been shown to have clinical relevance with regard to outcome under endocrine therapy.^22^, ^23^, ^24^ Effective endocrine therapy, either by hormone depletion or by receptor blockade, leads to growth arrest of tumor cells evidenced by a decrease in Ki67 labeling index. The Ki67 nuclear protein is expressed by cycling cells from G1 to M phase and provides a commonly used immunohistochemical method to assess the growth fraction in BC.^25^ A sustained high Ki67 index despite hormonal blockade is thought to identify ER‐independent tumor cell proliferation.^26^

In this study, luminal early BCs from the prospective WSG‐ADAPT trial which did not respond to short‐term preoperative endocrine therapy (pET) were analyzed for alterations of genes which had recently been implicated in endocrine resistance in metastatic BC including *ARID1A*, *CCND1*, *ERBB2*, *ESR1*, *FGFR1*, *PAK1*, *PIK3CA*, and *TP53*.^7^

## 2 MATERIALS AND METHODS

### 2.1 Cases and tumor tissue

Pre‐ and postmenopausal patients with ER‐ and/or PR‐positive, HER2‐negative early BC as determined by local pathologic assessment, were treated with short‐term pET in the West German Study Group (WSG) ADAPT trial (NCT01779206). The details on study design were published previously.^27^, ^28^ pET was applied for 3 weeks. Premenopausal women were mostly treated by tamoxifen and the majority (>90%) of postmenopausal women were treated by aromatase inhibitors (letrozole, anastrozole, or exemestane). All cases were subjected to central pathology review (MHH). Formalin‐fixed, paraffin‐embedded (FFPE) tumor tissue from the diagnostic core needle biopsies at baseline was submitted for recurrence score (RS) testing at the laboratory of Genomic Health Inc. The case characteristics are shown in Table 1.

**TABLE 1:** Characteristics of the study cohort

	All cases	pET	
		TAM cohort	AI cohort
	*n* = 622	*n* = 286	*n* = 334
Age at diagnosis			
Median (range) in years	54 (28–76)	47 (28–68)	62 (43–76)
pT stage			
pT1	371 (59.6)	166 (58.0)	204 (61.1)
pT2	223 (35.9)	109 (38.1)	113 (33.8)
pT3	24 (3.9)	9 (3.1)	15 (4.5)
pT4	2 (0.3)	0 (0.0)	2 (0.6)
n.a.	2 (0.3)	2 (0.7)	0 (0.0)
pN stage			
pN0	541 (87.0)	245 (85.7)	296 (88.6)
pN1+	79 (12.7)	39 (13.6)	38 (11.4)
n.a.	2 (0.3)	2 (0.7)	0 (0.0)
Histological grade, baseline			
G1	46 (7.4)	26 (9.1)	20 (6.0)
G2	399 (64.1)	180 (62.9)	218 (65.3)
G3	177 (28.5)	80 (28.0)	96 (28.7)
pET			
Tamoxifen	286 (46.0)	286 (100.0)	0 (0.0)
Aromatase inhibitors	334 (53.7)	0 (0.0)	334 (100.0)
n.a.	2 (0.3)	0 (0.0)	0 (0.0)
ER status, baseline			
Negative	1 (0.2)	0 (0.0)	1 (0.3)
Low expression	0 (0.0)	0 (0.0)	0 (0.0)
Positive	620 (99.6)	286 (100.0)	332 (99.7)
n.a.	1 (0.2)	0 (0.0)	1 (0.3)
ER status, post‐pET			
Negative	0 (0.0)	0 (0.0)	0 (0.0)
Low expression	1 (0.2)	0 (0.0)	1 (0.3)
Positive	620 (99.6)	285 (99.6)	333 (99.7)
n.a.	1 (0.2)	1 (0.4)	0 (0.0)
PR status, baseline			
Negative	46 (7.4)	11 (3.8)	35 (10.5)
Low expression	27 (4.3)	10 (3.5)	17 (5.1)
Positive	549 (88.3)	265 (92.7)	282 (84.4)
n.a.	0 (0.0)	0 (0.0)	0 (0.0)
PR status, post‐pET			
Negative	137 (22.0)	14 (4.9)	123 (36.8)
Low expression	65 (10.5)	13 (4.5)	51 (15.3)
Positive	420 (67.5)	259 (90.6)	160 (47.9)
n.a.	0 (0.0)	0 (0.0)	0 (0.0)
HER2 status (ASCO/CAP 2018), post‐pET			
0, 1+, 2+/FISH negative	613 (98.6)	281 (98.2)	330 (98.8)
2+/FISH‐positive, 3+/FISH‐positive	8 (1.3)	4 (1.4)	4 (1.2)
n.a.	1 (0.2)	1 (0.4)	0 (0.0)
Ki67, baseline			
0–9	72 (11.6)	32 (11.2)	40 (12.0)
10–19	244 (39.2)	117 (40.9)	127 (38.0)
20–34	222 (35.7)	100 (35.0)	121 (36.2)
35–100	84 (13.5)	37 (12.9)	46 (13.8)
Ki67, post‐pET			
0–9	327 (52.6)	86 (30.1)	241 (72.2)
10–19	186 (29.9)	121 (42.3)	63 (18.9)
20–34	87 (14.0)	60 (21.0)	27 (8.1)
35–100	22 (3.5)	19 (6.6)	3 (0.9)
Oncotype DX RS, baseline			
0–11	142 (22.8)	52 (18.2)	90 (26.9)
12–25	362 (58.2)	180 (62.9)	182 (54.5)
26–100	101 (16.2)	46 (16.1)	55 (16.5)
n.a.	17 (2.7)	8 (2.8)	7 (2.1)

Note

Unless otherwise stated, the values are given in the format *n* (%), with *n*, number of cases. Low expression (ER and PR status) is defined as 1%–9% positive cells.

Abbreviations: AI cohort, cases treated with aromatase inhibitors (letrozole, anastrozole, or exemestane); ER, estrogen receptor; FISH, fluorescence in situ hybridization; n.a., not available; pET, preoperative endocrine therapy; PR, progesterone receptor; RS, recurrence score; TAM cohort, cases treated with tamoxifen.

Tumor specimens correspond to *n* = 301 unselected consecutive BC cases from the run‐in phase of the WSG‐ADAPT trial.^28^ To increase the number of specimens, we also included *n* = 400 consecutive cases from the main phase of the WSG‐ADAPT trial. A total of *n* = 79 cases were excluded due to (i) missing Ki67 at baseline or post‐pET, (ii) unavailable tissue blocks (returned to local centers upon clinical request), (iii) divergent histological subtype at baseline and post‐pET, (iv) triple‐negative hormone receptor status, and (v) insufficient DNA amount and/or quality. The total number of specimens available for statistical analysis was *n* = 622 (Table 1).

### 2.2 Immunohistochemistry and fluorescence in situ hybridization

Immunohistochemistry (IHC) for estrogen receptor (ER), progesterone receptor (PR), HER2, and Ki67 was performed in the central reference pathology unit of the ADAPT trial (in the years 2012–2016; prospective assessment) using a Benchmark Ultra automated stainer (Ventana). Immunological reagents and central IHC scoring methods are summarized in the (Table S1).

Ki67 index assessment was supported by digital quantification as follows: First two experienced pathologists independently scored the Ki67 index by eyeballing in a minimum of 500 tumor cells (semiquantitative assessment, 5% increment steps). Next, Ki67‐positive tumor cell nuclei were quantified using the digital pathology platform iScan Coreo (Ventana) and Virtuoso quantification software (v5.3, Ventana) as described previously.^25^ Next, a consensus Ki67 index was defined based on the three evaluations (2x semiquantitative 1x Virtuoso). In most cases, the semiquantitative Ki67 index that was nearest to the digital Ki67 index was accepted as the definite consensus Ki67 index.

Endocrine proliferative response (EPR) was determined by the post‐pET consensus Ki67 index. The EPR was categorized in four categories corresponding to optimal (post‐pET Ki67 <10%) versus slightly, moderately, and severely impaired proliferative response (post‐pET Ki67 10%–19%, 20%–34%, and ≥35%, respectively) (Figure S1). These provisional cutoffs were chosen only for the present exploratory molecular analysis and aimed for an utmost stringent definition of an optimal EPR.

HER2 was scored as 0, 1+, 2+, or 3+, in accordance with the Dako HercepTest. BCs with a HER2 score of 2+ or 3+, were subjected to HER2 fluorescence in situ hybridization (FISH). FISH categorization was in accordance with the American Society of Clinical Oncology/College of American Pathologists (ASCO/CAP) 2018 guidelines.^27^, ^29^

### 2.3 DNA extraction

Depending on tumor size 6–8 sections (8 µm) were taken. Genomic DNA was extracted from FFPE specimens with the Maxwell^®^ RSC DNA FFPE Kit on a Maxwell^®^ RSC instrument (Promega) according to the manufacturer's recommendations. DNA concentration was quantified using a Qubit 2.0 fluorometer (Thermo Fisher Scientific) and the dsDNA high sensitivity kit (Thermo Fisher Scientific).

### 2.4 Mutational analysis

Within this study, we analyzed candidate endocrine resistance genes (*ARID1A*, *BRAF*, *ERBB2*, *ESR1*, *HRAS*, *KRAS*, *NRAS*, and *TP53*) that were identified by Razavi et al. by comparing alterations in ER‐positive metastatic BCs with primary tumors.^7^ In addition, *PIK3CA* mutation status was determined because of its potential as a target for selective inhibition.^30^ The *GATA3* mutation status, a potential marker for sensitivity toward aromatase inhibitors, was also included.^10^

Mutational analysis of matched resection specimen (post‐pET) from all cases was carried out retrospectively by next‐generation sequencing (NGS) in the years 2018–2020. Targeted sequencing was performed using two customized amplicon‐based panels. Library preparation was performed with Ion AmpliSeq™ Library Kit 2.0 (Thermo Fisher Scientific). For quantification of prepared libraries, the Ion Library TaqMan™ Quantitation Kit (Thermo Fisher Scientific) was used. Sequencing was performed on an Ion S5 instrument (Thermo Fisher Scientific). The first panel covered the complete protein‐coding sequence (as well as 10 base pairs of flanking intron sequence to cover the splice sites) of *ERBB2*, *ESR1*, *PIK3CA*, and *TP53*. Mean mapped reads per case was 141,732 (range 5,718–2,910,098) and mean depths per base was 9460 (range 55–191,139). The second panel covered the complete protein‐coding sequence of *ARID1A*, *BRAF*, *GATA3*, *HRAS*, *KRAS*, and *NRAS*. Mean mapped reads per case was 282,100 (range 33,486–7,149,110) and mean depths per base was 1,213 (range 163–29,306) (Table S2).

Evaluation of sequencing data and variant annotation was performed with the ANNOVAR software and database tools (http://annovar.openbioinformatics.org/en/latest/).^31^ Variants with unknown significance were predicted as deleterious, when they were considered as pathogenic in the following in silico prediction tools: MutationTaster, MutationAssessor, CADD, SIFT, and PolyPhen‐2.

Alterations in the *TP53* gene can have various consequences for the functionality of the mutant p53 protein. Functional classification of *TP53* variants was performed according to two different nomenclatures. These included *TP53* variant classification according the International Agency for Research on Cancer (IARC, classification as [partially] functional vs. non‐functional) and the classification according to Molina‐Vila et al. (classification as disruptive vs. non‐disruptive).^32^, ^33^, ^34^, ^35^

### 2.5 DNA copy number analysis

DNA copy number analysis of *CCND1*, *FGFR1*, and *PAK1* was performed using digital PCR (dPCR) in all tissue specimens with a tumor cell amount >50% (*n* = 199).

TaqMan™ Copy Number Assays (*CCND1* assay ID: Hs02559587_cn; *FGFR1* assay ID: Hs05052584_cn and *PAK1* assay ID: Hs01931361_cn; Thermo Fisher Scientific) were used with 15.0 ng of FFPE DNA input. If less than 15.0 ng of DNA was present, the maximum input volume of 7.13 µl was used. The range of DNA input was 2.21–15.0 ng. Two reference gene assays were used for each target assay to determine the found amplifications. For detection of *CCND1* and *PAK1* amplifications *RPPH1* (catalog no. 4403326; Thermo Fisher Scientific) and *PMP22* (customized; Thermo Fisher Scientific) were used as reference assays. The reference assays for *FGFR1* amplifications were *TERT* (catalog no. 4403315; Thermo Fisher Scientific) and *RPPH1*.

All reactions were performed with QuantStudio™ 3D Digital PCR System (Thermo Fisher Scientific), 3D PCR Master Mix v2 (Thermo Fisher Scientific), and 3D PCR 20K Chip Kit v2 (Thermo Fisher Scientific). PCRs were performed on a flat block thermocycler. The reaction conditions were as follows: hot start at 96°C for 10 min, annealing at 56°C for 2 min, and denaturation at 98°C for 30 s for a total of 39 cycles, followed by a final extension step at 60°C for 2 min. Data were analyzed with QuantStudio™ 3D AnalysisSuite™ Software (Version 3.1.6; Thermo Fisher Scientific). The threshold for amplification was a ratio of target‐gene calls to reference‐gene calls of 2.2. Within this study, only those cases that showed amplification with both reference assays are considered to be amplified (Table S2).

### 2.6 Statistics

For statistical evaluation of the association between genetic alterations and pathologic parameters, we focused on candidate genes with a mutation frequency of ≥2.5%. The two‐sided Fisher's exact test and the Chi‐squared test for trends were used for contingency analysis. The results were considered to be statistically significant if *p* ≤ 0.05.

Multivariate logistic regression was used to analyze the independent ability of *TP53* status (mut vs. wt and classification according to IARC, respectively), pET (AI vs. tamoxifen), pT stage (pT2 vs. pT1 and pT3/4 vs. pT1), pN stage (pN1+ vs. pN0), histological grade (G3 vs. G1/2), Oncotype DX RS (26–100 vs. 0–25), baseline Ki67 (10%–19% vs. <10%, 20%–34% vs. <10%, and ≥35% vs. <10%), baseline ER status (% expression, continuous variable), and baseline PR status (% expression, continuous variable) to predict impaired EPR (post‐pET Ki67 ≥10%). Statistical analysis was performed with GraphPad Prism software Version 5.00 (GraphPad Software) and Stata/IC Volume 16.1. (Stata Corp).

## 3 RESULTS

### 3.1 Case characteristics

We performed targeted sequencing of 622 HR+/HER2‐early BCs from patients, enrolled in the WSG‐ADAPT trial (NCT01779206). Tumor specimens corresponded to unselected consecutive cases from the run‐in phase of the WSG‐ADAPT trial.^28^ To increase the number of specimens, we also included consecutive cases from the main phase of the WSG‐ADAPT trial. Table 1 shows the characteristics of the study population included in the present molecular analysis.

### 3.2 Type and frequency of genetic alterations

Genetic alterations in candidate endocrine resistance genes (*ARID1A*, *BRAF*, *ERBB2*, *ESR1*, *HRAS*, *KRAS*, *NRAS*, and *TP53*) were determined by next‐generation sequencing in resection specimens following short‐term pET.^7^ *PIK3CA* was included because the therapeutic potential of PI3K inhibitors in *PIK3CA*‐mutated BCs.^30^ The *GATA3* mutation status, a potential marker for sensitivity toward AIs, was also included.^10^ To evaluate gene amplification as a potential mechanism of resistance toward endocrine therapy, copy number analysis by digital PCR (dPCR) was performed for *CCND1*, *FGFR1*, and *PAK1* in a subgroup of cases (*n* = 199).^7^ Besides this, overexpression and/or amplification of *ERBB2* were determined conventionally by immunohistochemistry and FISH in the context of the central pathology review.

**FIGURE 1:**
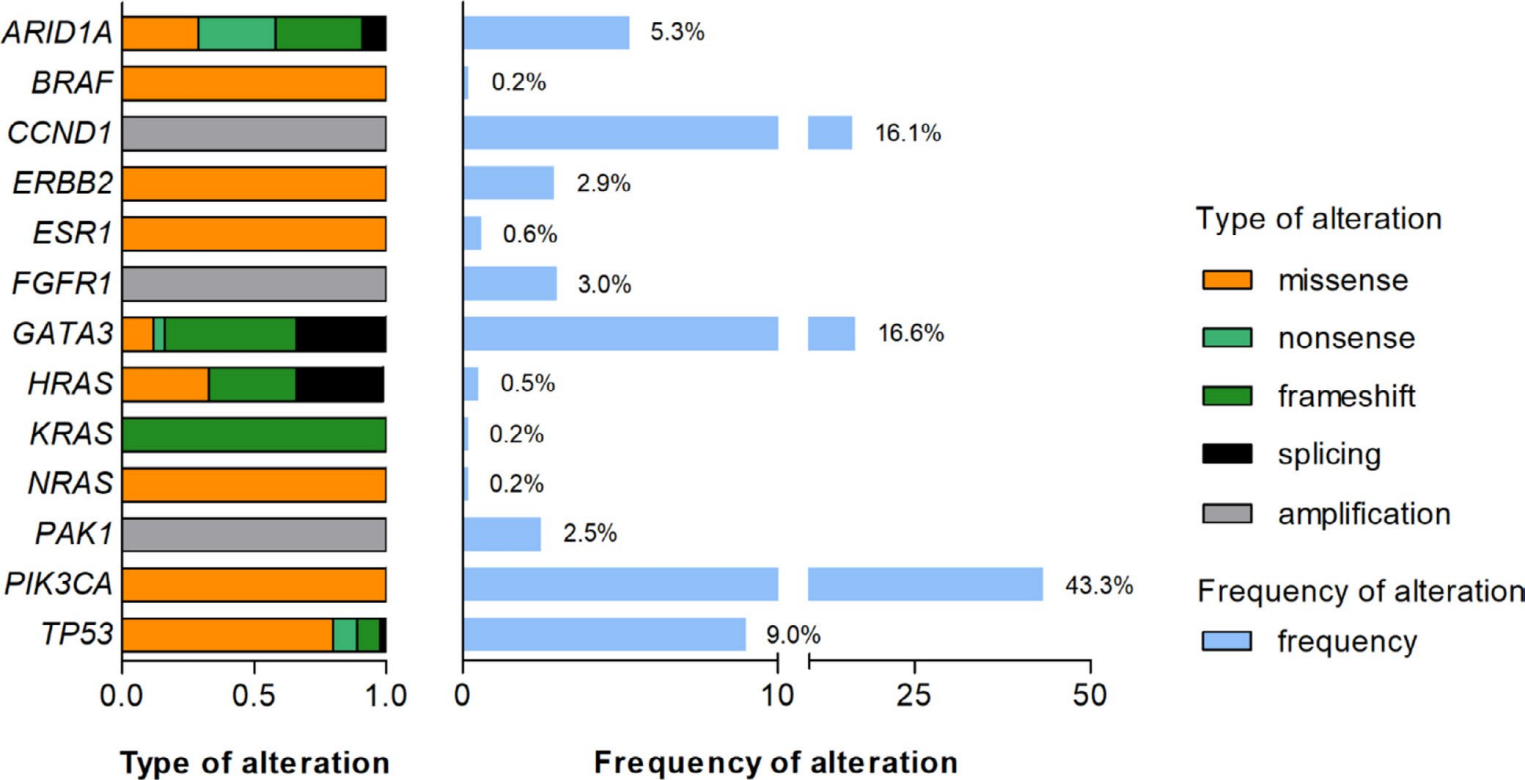
Histogram of the frequency and type of genetic alterations detected in this study

Somatic mutations or gene amplification were present in 66.2% (*n* = 412). A subset of these cases revealed more than one genetic alteration under study (18.3%, *n* = 114). Figure 1 shows an overview of the observed genetic alterations. Mutations of *ARID1A* were found in 5.3% of cases. Other somatic mutations with rates >5% of cases were *GATA3* (16.6%), *PIK3CA* (43.3%), and *TP53* (9.0%). Mutations of *BRAF*, *ERBB2*, *ESR1*, *HRAS*, *KRAS*, and *NRAS* occurred less frequently. Some genes like *PIK3CA* were mainly affected by missense mutations, whereas in other genes like *ARID1A* or *GATA3* truncating mutations were more frequent. In a total of 36 tumors (18.1%) *CCND1*, *FGFR1*, and/or *PAK1* amplifications were observed (Figure 1). Amplifications of *CCND1* (*n* = 32), *FGFR1* (*n* = 6), and *PAK1* (*n* = 5) were detected by dPCR in 16.1%, 3.0%, and 2.5% of cases under study, respectively (Figure 1). All tumors with *PAK1* amplification harbored a co‐amplification with *CCND1*. In addition, central pathology review revealed a positive HER2/*ERBB2* status (IHC 2+/FISH‐positive or IHC 3+/FISH‐positive) in a small subset of tumors (*n* = 8), which had been classified as HER2‐negative by local assessment (data not shown). These cases are termed *ERBB2*‐amplified herein. For subsequent statistical analysis, we focused on genes, which were altered in ≥2.5% of all cases and also included the *ERBB2*‐amplified tumors.

### 3.3 Association of genetic alterations with Oncotype DX RS

**FIGURE 2:**
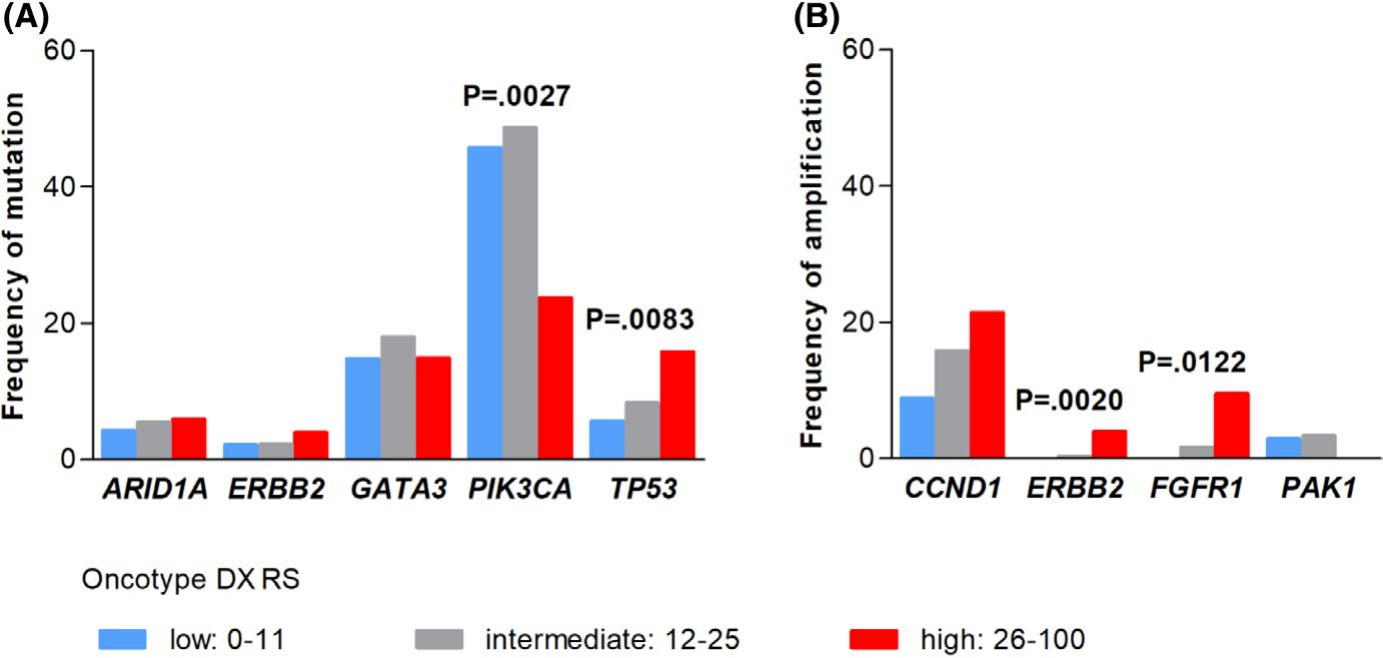
Distribution of alteration frequency according to Oncotype‐DX Recurrence Score (RS) groups. Gene mutations (A) and gene amplifications (B) are shown in two separate plots

For comparing the observed genetic alterations with the Oncotype DX RS, cases were grouped into three categories. In total, 22.8% (*n* = 142) were in the group with low RS (RS 0–11). The majority (58.2%, *n* = 362) had intermediate RS (RS 12–25) and 16.2% (*n* = 101) were categorized as high‐risk RS (RS ≥26). Figure 2A shows that *TP53* mutations were significantly more frequent in cases with high‐risk RS (15.8%, *n* = 16), than in cases with low RS 0–11 (5.6%, *n* = 8) (*p* = 0.0083). Nevertheless, in absolute numbers, the majority of *TP53* mutations were in the low and intermediate RS groups (Table S3). In contrast, *PIK3CA* mutations occurred significantly more frequent in cases with low RS (45.8%, *n* = 65) and were rarer in cases with high RS (23.8%, *n* = 24) (*p* = 0.0027). Mutations of *ARID1A*, *ERBB2*, and *GATA3* were equally distributed among RS groups (Figure 2A).

Amplification of *FGFR1* (0 to 9.5%) and *ERBB2* (0 to 4.0%) was significantly more frequent in cases with high RS (*p* = 0.0122 and *p* = 0.0020, respectively) (Figure 2B). *CCND1* amplifications were slightly more common in the high‐risk group, but this was not statistically significant. *PAK1* amplifications appeared to be equally distributed (Figure 2B).

### 3.4 Relation between genetic alterations and EPR

Endocrine proliferative response (EPR) to short‐term pET was determined by the post‐pET Ki67 index. In the present molecular analysis, EPR was categorized in four categories corresponding to optimal response (post‐pET Ki67 <10%) versus slightly, moderately, and severely impaired proliferative response (post‐pET Ki67 10%–19%, 20%–34%, and ≥35%, respectively) (Figure S1). In total, 52.6% of BC cases (*n* = 327) showed an optimal EPR (group I, median baseline Ki67 15.0%, median post‐pET Ki67 5.0%). In total, 29.9% of cases (*n* = 186) displayed a slightly impaired EPR (group II, median baseline Ki67 15.0%, median post‐pET Ki67 15.0%). In total, 14.0% and 3.5% of BC cases (*n* = 87 and *n* = 22) showed moderately and severely impaired EPR (group III and IV, median baseline Ki67 25.0% and 42.5%, median post‐pET Ki67 20.0% and 42.5%, respectively) (Figure S1).

**FIGURE 3:**
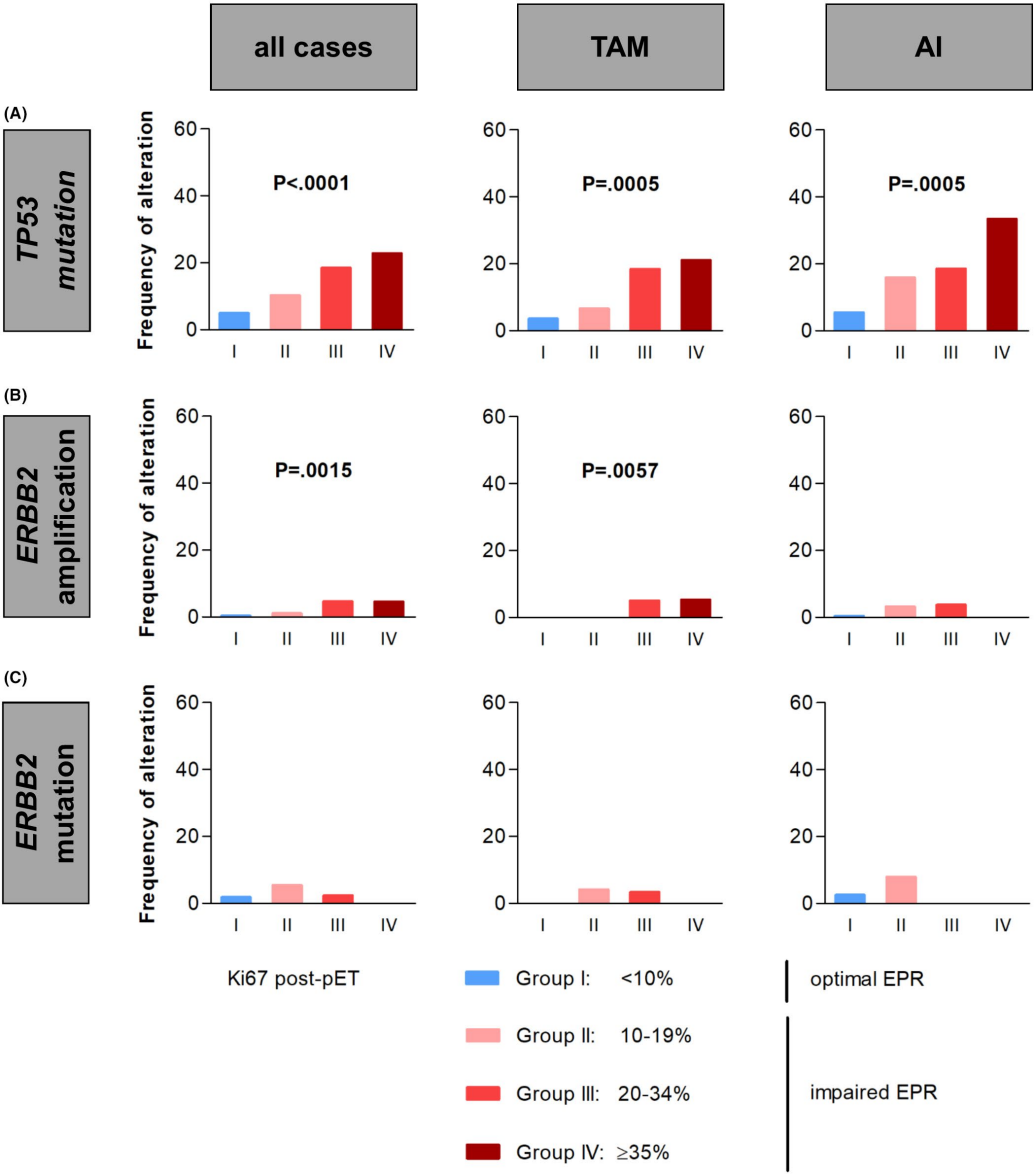
Relation between genetic alterations and endocrine proliferative response (EPR). Shown are the frequencies of *TP53*‐mutated breast cancer (BC) cases (A), *ERBB2*‐amplified BC cases (immunohistochemistry (IHC) 2+/fluorescence in situ hybridization (FISH)‐positive and IHC 3+/FISH‐positive, according ASCO 2018 guidelines) (B), and *ERBB2*‐mutated BC cases (C) according to post‐preoperative endocrine therapy (pET) Ki67 category. Subsets treated with either tamoxifen (TAM) or aromatase inhibitors (AI) are shown in the right panels

Two of the tested genetic alterations were significantly associated with impaired EPR (Figure 3). The frequency of *TP53* mutations increased significantly with increasing post‐pET Ki67 category. In detail, the frequency of *TP53* mutations increased from 4.9% in BC cases with optimal EPR (group I) to 22.7% in BCs with severely impaired EPR (group IV, *p* < 0.0001) (Figure 3A). This increase was independent from the mode of pET and occurred both with tamoxifen‐ as well as with AI‐based pET (*p* = 0.0005 each, Figure 3A).

**FIGURE 4:**
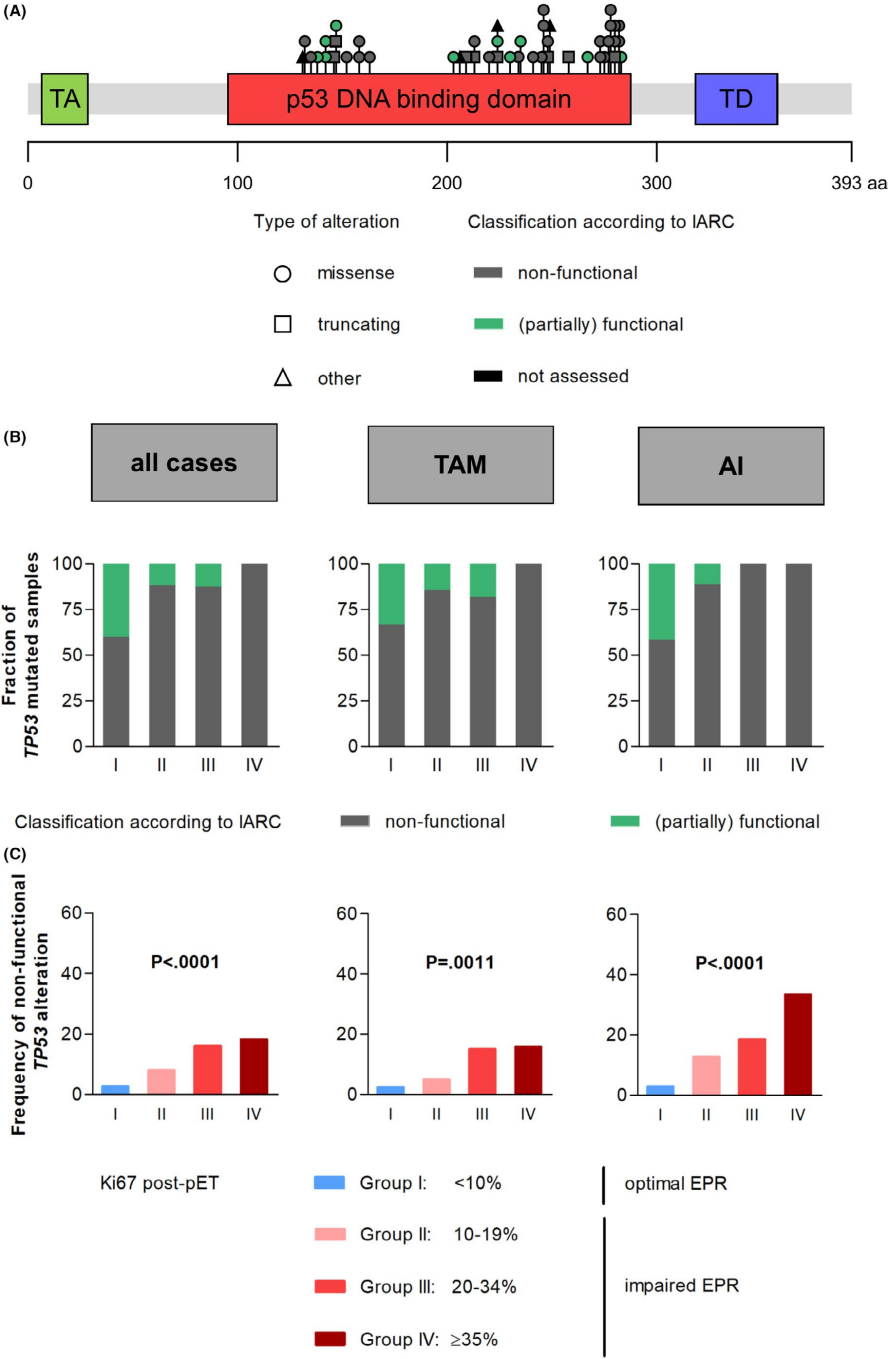
Relation between non‐functional *TP53* mutations and endocrine proliferative response (EPR). (A) The lollipop plot shows the distribution of mutations within the functional domains of the *TP53* gene. All alterations were observed in the DNA‐binding domain (aa 95–288). No alterations could be observed in the transactivation domain (TA, aa 6–29) and the tetramerization domain (TD, aa 318–358). (B) The bar chart shows the classification of mutations with the IARC *TP53* database according to post‐preoperative endocrine therapy (pET) Ki67 category. Variants that were not assessed were excluded for this illustration (splicing variants and in‐frame deletions, *n* = 3). (C) Relation between cases with non‐functional classified *TP53* mutations and EPR. Depicted are the frequencies according to post‐pET Ki67 category. For (B) and (C) subsets treated with either tamoxifen (TAM) or aromatase inhibitors (AI) are shown in the right panels

*TP53* mutations can be associated with variable loss of p53 transcriptional activity. According to IARC, the functional impact of *TP53* mutations is categorized as (partially) functional or non‐functional.^32^ Other classifications distinguish between non‐disruptive and disruptive *TP53* mutations.^34^, ^35^ Within this study, we detected 47 different *TP53* mutations in 56 patients. All detected *TP53* mutations were located in the p53 DNA‐binding domain (Figure 4A). Most of the detected mutations (77%; *n* = 43) were missense mutations. Truncating mutations were present in 18% (*n* = 10) of the patients and 5% (*n* = 3) harbored splicing mutations or in‐frame deletions. The majority of these *TP53* mutations (72%, 34/47) encoded for p53 mutants that are classified as non‐functional, according to IARC (Figure 4A). The minority of the detected *TP53* mutations was unclassified (6%, 3/47, including splicing mutations and in‐frame deletions), or (partially) functional (21%, 10/47), according to IARC (Figure 4A). The (partially) functional *TP53* mutations were more common in *TP53*‐mutated BCs with optimal EPR (group I) compared to *TP53*‐mutated BCs with severely impaired EPR (group IV, Figure 4B).

For completeness, we also conducted a refined statistical analysis considering the IARC classification to compare wild‐type *TP53* and *TP53* mutation encoding for a non‐functional p53 protein (Figure 4C). As expected, non‐functional *TP53* mutations were strongly associated with impaired EPR (Figure 4C). In detail, the frequency of non‐functional *TP53* mutations increased from 2.8% in BC cases with optimal EPR (group I) to 18.2% in BCs with severely impaired EPR (group IV, *p* < 0.0001, Figure 4C). This increase was independent from the mode of pET and occurred both with tamoxifen‐ as well as with AI‐based pET (*p* = 0.0011, respectively *p* < 0.0001, Figure 4C). Similar results were obtained for disruptive *TP53* mutations, as defined by Molina‐Vila et al. (Table S5).

In addition, tumors with positive HER2/*ERBB2* status (IHC 2+/FISH‐positive or IHC3+/FISH‐positive, as determined by central review) were also associated with EPR. Despite the small number of *ERBB2*‐amplified cases (*n* = 8) in this study, we observed a statistically significant correlation between *ERBB2* amplification status and impaired EPR (*p* = 0.0015, Figure 3B).

Activating mutation of the *ERBB2* gene occurred in *n* = 18/622 BC cases (all except one had a negative HER2/*ERBB2* status by IHC/FISH) and was not associated with impaired EPR (*p* = 0.6345, Figure 3C). Alterations of *ARID1A*, *CCND1*, *FGFR1*, *GATA3*, *PAK1*, and *PIK3CA* were not significantly associated with EPR (Table S4). However, *PIK3CA* mutation was associated with low baseline Ki67 and *TP53* mutation was also associated with high baseline Ki67 (Table S4).

### 3.5 *TP53* mutation is an independent predictive parameter for impaired EPR

**FIGURE 5:**
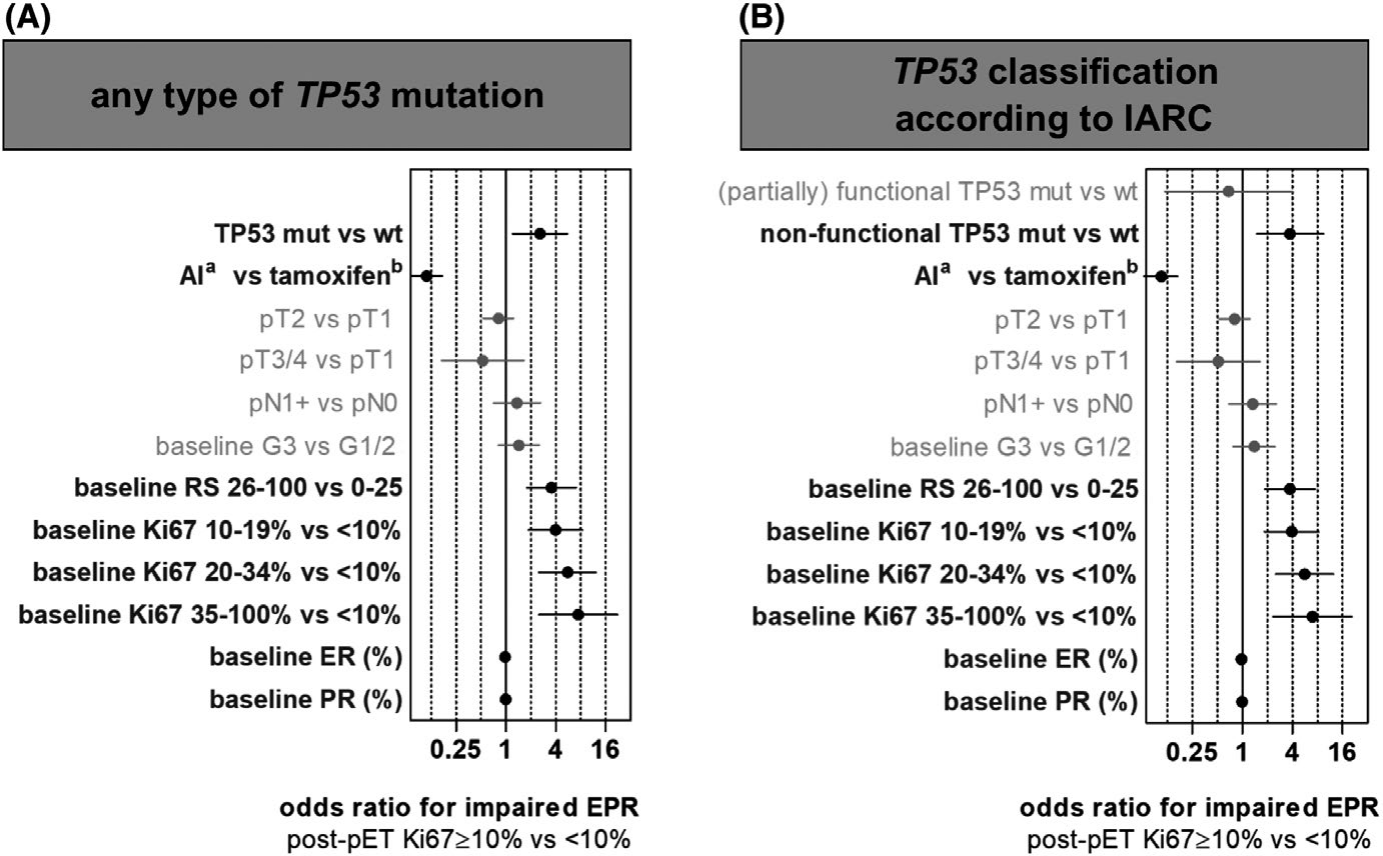
Multivariate logistic regression for the association of endocrine proliferative response (EPR) and multiple predictors. EPR was determined by the Ki67 index (Ki67 ≥10% vs. <10%) after preoperative endocrine therapy (pET). (A) Multivariate logistic regression for all cases with any type of *TP53* mutation versus *TP53* wild type. (B) Refined multivariate logistic regression considering the IARC classification for *TP53* mutation. Prognostic parameters were *TP53* status wild type (wt) and mutated (mut), pET (aromatase inhibitors (AI) and tamoxifen), pT stage, pN stage, baseline histological grade, baseline Oncotype DX Recurrence Score (RS) group, baseline Ki67, baseline estrogen receptor status (ER), and baseline progesterone receptor status (PR). ^a^Mostly postmenopausal; ^b^mostly premenopausal

Using multivariate logistic regression, we analyzed multiple factors for their ability to predict impaired EPR (Figure 5). To this end, we focused on the following parameters such as *TP53* mutation status, type of pET (tamoxifen vs. AI), pT stage, pN stage, baseline histological grade, Oncotype DX RS group, baseline Ki67, baseline ER status (% expression, continuous variable), and baseline PR status (% expression, continuous variable). Independent predictive parameters for impaired EPR included *TP53* mutation status, type of pET, RS group, baseline Ki67 index, baseline ER status, and baseline PR status (Figure 5).

Tumors with *TP53* mutations (any type) had higher odds of impaired EPR (OR = 2.6, 95% CI = 1.2–5.5) compared to *TP53* wild‐type tumors. Risk of impaired EPR was 3.5 times higher in cases with RS 26–100, than in cases with RS 0–25. Cases with high baseline Ki67 also had higher odds for an impaired EPR. pT stage, pN stage, and histological grade did not independently predict impaired EPR (Figure 5A, Table S6).

For completeness, we used a refined multivariate logistic regression considering the IARC classification to compare wild‐type *TP53* and non‐functional *TP53* mutations (Figure 5B). In this refined analysis, risk of impaired EPR was 3.7 times higher in tumors with *TP53* mutations (95% CI = 1.5–9.4), than in tumors without *TP53* mutations. Hence, *TP53* mutation predicts impaired EPR independently from Oncotype DX RS and other clinicopathological parameters.

## 4 DISCUSSION

The greatest part of our knowledge regarding endocrine resistance in BC stems from experimental in vitro studies using cell lines and animal models. Over the last decades, tamoxifen sensitivity has been the subject of many, primarily cell biological studies, but none of the many potential markers had found its way into the clinic. The major reason for this deficit in translation is the lack of clinical studies suitable to validate the in vitro findings.^36^

A novel approach to understand endocrine resistance was enabled by large‐scale mutational analysis of relapsing and metastatic BC which had been treated with adjuvant endocrine therapy after surgery.^7^, ^13^ In the study of Razavi et al. including 1500 luminal BC, the majority of cases had been exposed to prior therapy (87.5% of the biopsied metastatic tumors).^7^ Also, the tumors which were entered into the study of Bertucci et al. had received prior cytotoxic treatment.^12^ Treatment interferes with clonal evolution and selection of subclones in BC as has been obvious from *ESR1* mutation which is far more frequent in previously AI‐treated luminal cancers than in treatment‐naïve cases.^37^ Whole exome sequencing of 507 primary BC showed that only three gene mutations occurred at >10% incidence across all BCs, these were *GATA3*, *PIK3CA*, and *TP53*.^8^ Overall *TP53* mutation was found in 37% of BC, with a frequency of 12% in luminal A, and 29% in luminal B type of BC, respectively.^8^ Interestingly, luminal/ER‐positive BC proved to be the most heterogeneous in terms of gene expression, mutation spectrum, copy number variations, and outcomes.^8^

Despite the huge number of BC cases which have undergone next‐generation sequencing, there are no prospective clinical trials in primary, non‐metastatic BC which exploit the available bulk of sequence data in order to stratify cases according to their endocrine responsiveness. Currently, gene expression profiles like Oncotype DX RS are utilized to achieve this goal.^38^ There appear to be two reasons to explain the missing utilization of available DNA sequence data for this purpose. One reason is provided by the striking heterogeneity of primary BC, in particular luminal cancers.^8^ Second, in particular with regard to the recent studies on metastatic cancer, there is no certainty whether the numerous genetic alterations discovered represent primary or secondary aberrations induced by or selected during treatment.^7^

With regard to luminal BC, identification of the resistant sub‐cohort by impaired EPR after pET could enable recognition of primary aberrations associated with endocrine resistance. In previous smaller studies which have used this approach, *TP53* mutation has not been clearly identified as a potential source of endocrine resistance.^10^, ^17^, ^39^, ^40^ Previously, Gellert et al. have hypothesized that poor responders were more likely to have *TP53* mutations compared with good responders.^17^ However, they had to reject this hypothesis after statistical analysis of their limited tumor collection from the POETIC clinical trial (*n* = 66, their *p* = 0.8).^17^ Ellis et al. reported correlations between *TP53* mutations and higher Ki67 levels at baseline and at surgery, as well as a correlation with the luminal B subtype.^10^ In AI non‐responders they found an increased prevalence of genetic alterations in p53 signaling pathway (including *TP53*, *ATR*, *APAF1*, and *THBS1* combined), but predictive relevance of *TP53* mutations per se was not evaluated.^10^ Giltnane et al. could not find any correlation between *TP53* mutation and impaired EPR in a limited tumor collection from a clinical trial at the Vanderbilt University (*n* = 140).^39^ Using gene expression profiling Gao et al. showed that a *TP53* dysfunction signature was associated with impaired EPR after 2 weeks of pET with AI, but *TP53* gene mutation status was unknown.^40^ Consequently, *TP53* mutation is currently not considered as a determinant of endocrine resistance.^1^ In the WSG‐ADAPT trial, there was, for the first time, a clear‐cut association between impaired EPR and *TP53* mutation, in AI‐ as well as tamoxifen‐treated BC. Different reasons may account for the fact that *TP53* mutation was associated with EPR in our study, but not in previously reported studies. One possible reason is the considerably larger sample sizes in the present study.^17^, ^39^, ^40^ Another possible reason is that previous studies had included HER2‐positive along with HER2‐negative HR‐positive BCs.^10^, ^17^, ^40^ In our study, HER2‐positive BCs were almost completely excluded as per clinical study inclusion criteria. A third reason might be different criteria to define EPR.^10^, ^17^, ^39^, ^40^ In the present study, impaired EPR was defined as post‐pET Ki67 ≥10% and optimal EPR was defined as post‐pET Ki67 <10%. This is consistent with the definition of EPR in a recent analysis of the POETIC trial.^24^ EPR cutoffs utilized in previous studies varied between 2.7% and 10%.^10^, ^39^ Moreover, *TP53* analysis was based on slightly different methods.^10^, ^40^ In one study, *TP53* mutation status was assumed based on a gene expression signature.^40^ In the present study, the complete coding sequence of the *TP53* gene was analyzed for mutations by NGS.

The overall frequency of *TP53* mutations in the BC collection analyzed in this study was comparatively low (9%).^8^ However, this may be related to the characteristics of BC patients preferably enrolled in the WSG‐ADAPT HR‐positive /HER2‐negative trial. The frequencies of other genetic alterations in our cohort were mostly very similar to published data for primary BC.^8^, ^39^, ^41^ With 0.6% instead of 3%, we found fewer *ESR1* mutations than expected.^36^

There have been conflicting data regarding *TP53* mutation as a predictive marker in BC, indicating either increased sensitivity to cytotoxic drugs or on the contrary potential resistance.^16^ Analyzing the METABRIC data, the influence of *TP53* mutation on survival of BC cases was studied. Thereby, two effects of the mutation could be observed. First, *TP53* mutant cancers displayed a superior overall survival when treated by chemotherapy and irradiation. Second, *TP53* wild‐type cases revealed substantial benefit when ER‐expressing cancers were treated with endocrine therapy. Overall survival in this study was worse in endocrine‐treated cases with *TP53* mutation than in *TP53* wild‐type cancers, corresponding to our findings that *TP53* might be an effector of endocrine resistance.^16^ Further studies are warranted to corroborate the relationship between *TP53* and endocrine responsiveness in more detail. In vitro cell models of HR+BC cell lines harboring *TP53* mutations introduced by the CRISPR‐Cas9 method might help to clarify whether or not sensitivity to estrogen deprivation is directly dependent on the *TP53* status. Furthermore, large‐scale sequencing studies in clinical cohorts with long‐term follow‐up may document the relevance of *TP53* mutation for primary endocrine resistance.

Besides of *TP53* mutation, only RS group, type of pET (associated with age), and baseline Ki67 were associated with impaired EPR. For recurrence score, Paik et al. have similar findings in cases treated with tamoxifen.^42^ The POETIC trial showed that short‐term pET with AIs (2 weeks of letrozole or anastrozole) does not improve outcome (BC recurrence). However, it was also shown that cases with high Ki67 at baseline and after preoperative therapy have a higher risk of recurrence.^24^ pET could be used to select an appropriate adjuvant therapy based on the observed Ki67 response, because impaired EPR might be an early indication for primary endocrine resistance.^24^ Unfortunately, we did not find associations between impaired EPR with targetable alterations, like *FGFR1* amplifications and *PIK3CA* mutations.

In conclusion, the current WSG‐ADAPT translational study demonstrates that impaired EPR is suitable to identify genetic mechanisms of primary endocrine resistance already early during the course of early luminal BC. The presence of *TP53* mutations indicates primary endocrine resistance in about 10% of luminal early BC cases. As *TP53* mutations have also been implicated with conveying sensitivity toward conventional chemotherapy, further studies are needed in order to clarify the clinical consequences of our findings.

## CONFLICT OF INTEREST

OG has minority ownership interest in WSG GmbH, received honoraria from Genomic Health/Exact Sciences, Roche, Celgene, Pfizer, Novartis, NanoString Technologies, AstraZeneca, served in consulting/advisory role for Celgene, Genomic Health/Exact Sciences, Lilly, MSD, Novartis, Pfizer, Roche, and received travel support from Roche.

UN has minority ownership interest in WSG GmbH, received honoraria from Agendia, Amgen, Celgene, Genomic Health, NanoString Technologies, Novartis pharma, Pfizer Pharmaceuticals, Roche/Genentech, Teva, served in consulting/advisory role for Genomic Health, Roche, provided expert testimony for Genomic Health, received travel support from Genomic Health, Pfizer Pharmaceuticals, Roche, and her institution received research funding from Agendia, Amgen, Celgene, Genomic Health, NanoString Technologies, Roche, Sanofi.

SK has minority ownership interest in WSG GmbH, received personal fees from Lilly, Roche, Genomic Health, Novartis, Amgen, Celgene, Daiichi Sankyo, AstraZeneca, SOMATEX Medical Technologies, MSD, Pfizer, Puma Biotechnology, PFM medical, and non‐financial support from Roche, Daiichi Sankyo, Sonoscope.

MB received honoraria from AstraZeneca, Exact Sciences, Novartis, Pfizer, Roche, Teva, travel support from AstraZeneca, Celgene, Medac, Novartis, Roche and served in consulting/advisory role for AstraZeneca, Exact Sciences, Novartis, Puma, Roche.

BA reports a potential financial conflict of interest as follows: Pfizer Pharma GmbH, Roche Pharma AG, Novartis Pharma GmbH, AstraZeneca GmbH, PharmaMar GmbH, MSD Merck Sharp & Dohme GmbH, Onkowissen.de GmbH, Lilly Deutschland GmbH, ProMedicis GmbH.

RK served in consulting/advisory role for West German Study Group, and reports for an immediate family member: ownership interest in WSG GmbH, honoraria from Amgen, AstraZeneca, Genomic Health, Novartis, Pfizer, Pierre Fabre, Roche, Zodiac Pharma, consulting/advisory role for Agendia, AstraZeneca, Celgene, Daiichi Sankyo, Lilly, Merck Sharp & Dohme, Novartis, Odonate Therapeutics, Pfizer, Pierre Fabre, Roche/Genentech, Sandoz, Seattle Genetics, and research funding from Lilly, Merck Sharp & Dohme, Novartis, Pfizer, Roche/Genentech.

RW served in consulting/advisory role as well as on speakers’ bureau for and received travel support from Agendia, Amgen, Aristo, AstraZeneca, Boehringer Ingelheim, Carl Zeiss, Celgene, Clinsol, Daiichi‐Sankyo, Eisai, Genomic Health, Glaxo Smith Kline, Hexal, Lilly, Medstrom Medical, MSD, Mundipharma, NanoString, Novartis, Odonate, Onkowissen, Paxman, Palleos, Pfizer, Pierre Fabre, Puma Biotechnology, Riemser, Roche, Sandoz/Hexal, Seattle Genetics, Tesaro Bio, Teva, Viatris.

NH has minority ownership interest in WSG GmbH, received honoraria from Amgen, AstraZeneca, Genomic Health, Novartis, Pfizer, Pierre Fabre, Roche, Zodiac Pharma, served in consulting/advisory role for Agendia, AstraZeneca, Celgene, Daiichi Sankyo, Lilly, Merck Sharp & Dohme, Novartis, Odonate Therapeutics, Pfizer, Pierre Fabre, Roche/Genentech, Sandoz, Seattle Genetics, an immediate family member served in consulting/advisory role for West German Study Group, and her institution received research funding from Lilly, Merck Sharp & Dohme, Novartis, Pfizer, Roche/Genentech.

IG, SB, LK, LB, HC, MGro, MR, UL, CzE, EMG, CS, KLH, MGra, MC, and HK, declare no potential conflict of interest.

## ETHICAL APPROVAL STATEMENT

The study design is following the guidelines of the local ethics committee (“Ethics committee of the Medical School Hannover/Ethik‐Kommission der Medizinischen Hochschule Hannover,” head: Prof. Dr. Albrecht).

## Supporting information

Figure S1

Table S1

Table S2

Table S3

Table S4

Table S5

Table S6

## Data Availability

Data generated or analyzed during this study are included in this published article and its additional files.
