# Supplementary figures and images for: TP53 mutations are associated with primary endocrine resistance in luminal early breast cancer
Source: Cancer Med. 2021 Nov 14;10(23):8581–94. doi: 10.1002/cam4.4376 (PMC8633262; doi:10.1002/cam4.4376)

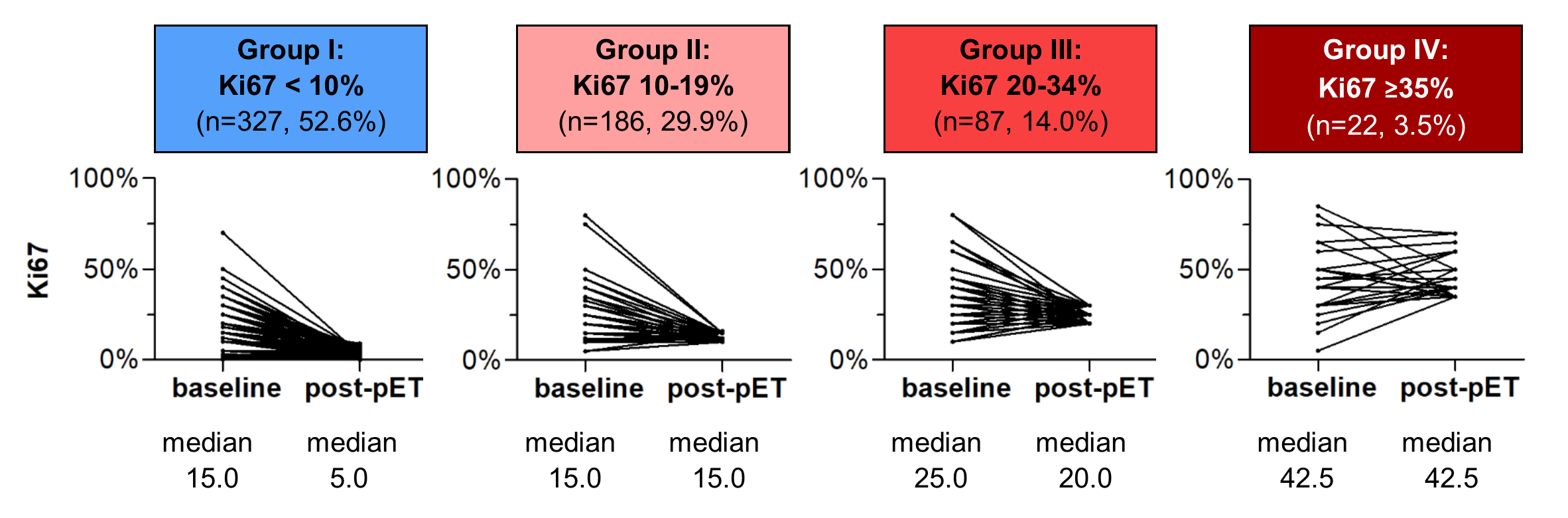

Supplement: Supplementary file 1 — Figure S1 [file CAM4-10-8581-s006.tif]
